# Sleep predicts the response to rTMS and CBT in patients with OCD: an open label effectiveness study

**DOI:** 10.1016/j.ijchp.2022.100353

**Published:** 2022-11-15

**Authors:** Priya T. Gajadien, Tjardo S. Postma, Iris van Oostrom, Karel W.F. Scheepstra, Hanneke van Dijk, Alexander T. Sack, Odile A. van den Heuvel, Martijn Arns

**Affiliations:** a Universiteit van Amsterdam, the Netherlands; b Research Institute Brainclinics, Brainclinics Foundation, Nijmegen, the Netherlands; c Department of Psychiatry and Department of Anatomy & Neuroscience, Amsterdam UMC, Vrije Universiteit Amsterdam, Amsterdam Neuroscience (Compulsivity, Impulsivity and Attention), Amsterdam, The Netherlands.; d Faculty of Psychology and Neuroscience, Maastricht University, the Netherlands; e Amsterdam UMC, University of Amsterdam, Adult Psychiatry, Amsterdam Neuroscience, Meibergdreef 9, Amsterdam, Netherlands; f Neuroimmunology research group, Netherlands Institute for Neuroscience, Meibergdreef 47, 1105 BA, Amsterdam, the Netherlands; g neurocare clinics, Nijmegen, the Netherlands

## Abstract

**Background:**

Although many OCD patients benefit from repetitive transcranial magnetic stimulation (rTMS) as treatment, there is still a large group failing to achieve satisfactory response. Sleep problems have been considered transdiagnostic risk factors for psychiatric disorders, and prior work has shown comorbid sleep problems in OCD to be associated with non-response to rTMS in OCD. We therefore set out to investigate the utility of sleep problems in predicting response to rTMS in treatment resistant OCD.

**Method:**

A sample of 61 patients (treated with 1-Hz SMA or sequential 1-Hz SMA+DLPFC rTMS, combined with cognitive behavioral therapy) were included. Sleep disturbances were measured using the PSQI, HSDQ and actigraphy. Treatment response was defined as a decrease of at least 35% in symptom severity as measured with the Yale-Brown Obsessive-Compulsive Scale (Y-BOCS).

**Results:**

32 of 61 patients (52.5%) responded to rTMS, and trajectories of response were similar for both rTMS protocols. Three PSQI items (Subjective Sleep Quality; Sleep Latency and Daytime Dysfunction) and the HSDQ-insomnia scale were found to predict TMS response. A discriminant model yielded a significant model, with an area under the curve of 0.813.

**Conclusion:**

Future replication of these predictors could aid in a more personalized treatment for OCD.

## Introduction

Obsessive-compulsive disorder (OCD) is a common, severe psychiatric disorder affecting 2-3% of the world population (Stein et al., 2019). It is characterized by intrusive thoughts or images (obsessions) and repetitive ritualistic behaviors to avoid or reduce distress (compulsions) (American Psychiatric Association, 2013). OCD frequently follows a chronic course and results in impairment and a decrease of quality of life in many domains (Subramaniam et al., 2013). Although first line treatment strategies including exposure-based cognitive-behavioral therapy (CBT) and serotonergic antidepressants can be reasonably successful for a substantial group of patients, 40-50% of OCD patients are not sufficiently benefitting from these therapies (Denys, 2006; Heyman et al., 2006).

As an alternative treatment approach, repetitive transcranial magnetic stimulation (rTMS) holds promise in alleviating symptoms in OCD (Fitzsimmons et al., 2022). This non-invasive neuromodulation technique employs a strong pulsating magnetic field over the scalp, inducing electrical currents in neurons of the underlying cortex, changing cortical excitability at the targeted brain region as well as connected remote areas within functional neural networks (Barker et al., 1985). Repetitive TMS could be used to target specific brain areas within neural networks involved in OCD, related to emotional emotion regulation, response inhibition and other aspects of cognitive control on emotion and behavior (Stein et al., 2019).

Several studies demonstrated the importance of the cortico-striato-thalamo-cortical (CSTC) circuit as the prevailing neurobiological model of OCD neurobiology (Dougherty et al., 2020; Nakao et al., 2014). The CSTC theory states that an imbalance between and within these pathways may result in maladaptive behavior as seen in OCD (Shephard et al., 2021; Stein et al., 2019). Two promising cortical areas involved in the CSTC that are often selected as a target for rTMS are the pre-supplementary motor area (SMA) and the dorsolateral prefrontal cortex (DLPFC) and in a recent meta-analysis Fitzsimmons et al. (2022) reported that rTMS aimed at both the SMA and the DLPFC were efficacious relative to sham for OCD treatment with a medium effect size. Despite these promising results regarding the therapeutic potential of rTMS for the treatment of OCD, many patients still fail to respond, making it vital to be able to predict treatment (non-)response by investigating potential (bio)markers.

In a prior pilot study, we demonstrated promising clinical results in an open-label setting with a 55% response rate for combined rTMS and psychotherapy, as well as a higher prevalence of sleep problems in the OCD population compared to controls and evidence for circadian rhythm sleep problems (CRSD) to be strongly associated with non-response to rTMS (Donse et al., 2017). Given that sleep problems are part of the diagnostic criteria of most DSM 5 disorders, sleep problems can be considered a trans-diagnostic feature, either causally related to specific symptoms such as inattention or mood, or as exacerbating factors (Arns et al., 2021).

As some of these sleep problems could be relatively easy to treat with, for example, light-therapy for CRSD or sleep hygiene and Cognitive Behavioral Therapy for insomnia (CBT-I), this calls for further research to investigate the role of sleep problems as predictors for treatment response (Arns et al., 2021). Therefore, the aims of the present study were (1) to investigate differences between responders and non-responders to rTMS on sleep parameters and (2) establish the potential predictive value of these variables for rTMS response in OCD.

## Methods

### Patients

Sixty-one patients (mean age: 37.83 ± 13.60, 43 males) with a primary diagnosis of OCD who were treated with rTMS were included (20 of these patients were also reported in Donse et al., 2017). All patients were recruited for treatment at three neuroCare Group clinics (Nijmegen, Den Haag, and Eindhoven, the Netherlands) between June 2013 and April 2021 and provided written informed consent. The primary diagnosis of OCD was confirmed by a licensed clinical psychologist, according to the Mini-International Neuropsychiatric Interview (M.I.N.I.) or Structured Clinical Interview for DSM-5 the (SCID-5-S) and a score ≥16 on the Yale-Brown Obsessive-Compulsive Screening (Y-BOCS). OCD patients were only offered rTMS when no clear clinical response on prior CBT and/or medication was achieved. The majority of patients had a primary diagnosis of OCD (55.7%), followed by comorbidity with depression/dysthymia (39.3%), anxiety (34.4%), somatoform disorder (8.2%) and post-traumatic stress disorder (3.3%). The majority of patients were using medication, medication consisted of selective serotonergic re-uptake inhibitors (SSRI; 36.1%), benzodiazepines (22.6%), tricyclic antidepressants (TCA; 11.5%) and/or antipsychotics (11.5%). According to the safety criteria for rTMS, patients with a pacemaker or metals in the head area, pregnancy and a presence or a history of epilepsy were excluded (Rossi et al., 2009).

### Design

The current study was a naturalistic open-label study, with patients receiving treatment as usual. Treatment outcome was determined for patients with a treatment course of at least 10 rTMS sessions. Patients with a full treatment received either a low frequency (LF) SMA protocol (n=35) or a LF SMA+DLPFC protocol (n=26) both combined with CBT during rTMS. All rTMS was delivered using standard figure-8 coils, and combined LF SMA+DLPFC protocols were conducted sequentially, with approximately five minutes in between for changing the coil position.

### Treatment procedure

The SMA protocol was primarily indicated for treatment of primary OCD symptoms. However, in case of a comorbid depression as determined by the licensed clinical psychologist based on DSM-IV/5 criteria a sequential double protocol was indicated, where – within one session - 1-Hz rTMS aimed at SMA (1200 pulses, 110% MT, SMA at 15% of the distance between nasion and inion anterior to the vertex (Cz) (Mantovani et al., 2010), was combined with 1-Hz rTMS aimed at the right DLPFC (1000-1200 pulses, 120% MT, BEAM-F3 method (Beam et al., 2009). Number of pulses for DLPFC rTMS was standard 1200 pulses, except in a minority of situations where overheating issues in patients with high MT's prompted the clinician to reduce the number. Treatment response was assessed with the Y-BOCS after each 5^th^ session. Each session had a total duration of 45-60 minutes including cognitive behavioral therapy (CBT) and rTMS. CBT consisted mainly of exposure with response prevention, frequently complemented with additional CBT tailored to the individual patient. Patients were treated for at least two times a week with a maximum of five times a week.

Before the start of the rTMS treatment, the motor threshold (MT) was established at the intensity required for producing a motor evoked potential (MEP) in the contralateral abductor pollicis brevis to exceed a defined peak-to-peak amplitude in 50% of the pulses.

### OCD, Mood and sleep assessments

The Y-BOCS was used as primary outcome measure from intake to outtake. Remission was defined as a score of ≤12, and response as a decrease of ≥ 35% on the Y-BOCS from baseline to post-treatment (Mataix-Cols et al., 2016). For further prediction analyses we relied primarily on response status. To track depressive symptoms, the Beck Depression Inventory, second edition, Dutch version (BDI-II-NL) questionnaire was used. Sleep disturbances were investigated using the self-rating questionnaires Pittsburg Sleep Quality Index (PSQI) and Holland Sleep Disorder Questionnaire (HSDQ), and an actigraphy watch (Condor, ActTrust) that objectively measures sleep-wake and activity was assessed for at least 7 days prior to treatment.

### Statistics

All analyses were performed using IBM SPSS Statistics 27. Differences in age and sex were tested using a One-Way ANOVA and a Chi-square. The main treatment outcome analysis consisted of repeated-measures analysis of variance (ANOVAs) with within subject factor Time (pre- and post-treatment) and between subject factor Protocol (SMA/SMA+DLPFC rTMS) to test changes over time in OCD patients in Y-BOCS and BDI scores. Significant effects were complemented with Cohen's *d* effect size.

Further analyses focused on rTMS treatment outcome and predictors of rTMS response using discriminant analysis. First, One-Way ANOVA's were conducted on baseline sleep variables between responders and non-responders and correlations established between sleep variables and percentage improvement on the YBOCS. Next, sleep parameters with an effect size *d*>0.5 were defined as potential predictors and entered into the discriminant analysis. If variables demonstrated collinearity with r>0.7, the collinear variable was not included into the discriminant analysis. Receiver Operator Curves (ROC) were established and area under the curve (AUC) reported as well as sensitivity, specificity, positive predictive value and negative predictive value.

## Results

### Demographics

The sample consisted of 61 OCD patients, 35 treated with a single protocol (1-Hz SMA; age: 38.32 ± 13.27, 17 males) and 26 patients treated with a double protocol (1-Hz SMA + 1-Hz DLPFC; age: 37.00 ± 13.55, 17 males). Patient demographics with treatment outcomes are described in Table 1. In the total sample, no differences in age (F(1,59) = 0.15, *p* = .704) or sex (χ^2^(1,n = 61) = 1.71, *p* = .191) were detected between patients with a single and a double protocol. Therefore, age and sex were not considered as covariates in the analysis. At baseline, OCD symptom severity revealed no significant differences between protocols (F(1,59) = 1.43, *p* = .236, *d* = .31). As a result of the treatment allocation, the patients in de double protocol had significantly higher severity of depressive symptoms at baseline (F(1,56) = 13.93, *p* < .001).

**Table 1:** Demographic features of OCD patients, treatment outcomes per protocol and baseline behavioral scores of OCD symptoms and depressive symptoms.

	All (n=61)	1-Hz SMA (n=35)	1-Hz SMA + 1-Hz DLPFC (n=26)
Age (y)	37.76 (13.29)	38.32 (13.27)	37.00 (13.55)
Males (n (%))	34 (55.7)	17 (48.6)	17 (65.4)
Medicated (n (%))	42 (68.9)	25 (71.4)	17 (65.4)
Responders (n (%))	32 (52.5)	21 (60.0)	11 (42.3)
Remission (n (%))	18 (29.5)	13 (37.1)	4 (15.4)
Number sessions (mean, sd)	27.8 (10.0)	28.5 (9.8)	26.7 (10.5)
Y-BOCS pre-treatment (mean, sd)	27.25 (6.21)	26.43 (6.40)	28.35 (5.90)
BDI pre-treatment (mean, sd)*	25.12 (10.9)	20.94 (10.2)	30.64 (9.3)

*For the BDI (n=57), 25 patients were treated with a 1-Hz SMA protocol and 33 patients were treated with a 1-Hz SMA + 1-Hz DLPFC protocol.

### Treatment outcome

Repeated-measures ANOVAs (Group x Protocol) demonstrated a significant effect of Time with a large effect size for OCD symptoms in the total group (*F*(1,59) = 73.89, *p* < .001, *d* = 1.37), and Protocol (F(1,59) = 5.92, *p* = .018), but no significant Time X Protocol interaction (F(1,59) = 2.31, *p* = .134).

**Fig. 1:**
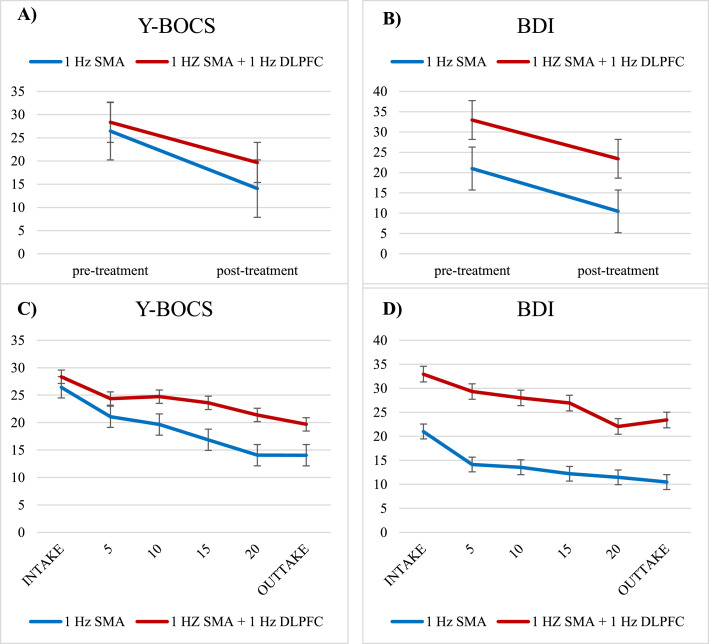
Representation of the time course of symptom change for 1-Hz SMA (Blue) and 1-Hz SMA + 1-Hz DLPFC (Red) protocols over the course of treatment for OCD symptoms (Y-BOCS) and depressive symptoms (BDI). Figure 1A and 1B visualize all data included in the statistical tests (Y-BOCS, N=61 and BDI, N=54), figure 1C and 1D visualize the same time course using a more fine-grained time-course for visualization purposes with symptoms assessed every 5^th^ session (albeit with a smaller sample size, Y-BOCS, N=42, BDI, N=46). Error bars represent SEM.

For depressive symptoms, repeated-measures ANOVAs demonstrated a significant effect of Time with a large effect size (*F*(1,52) = 33.14, *p* < .001, *d* = .80) and Protocol (F(1,52) = 23.81, *p* < .001), but no Time X Protocol interaction (F(1,52) = 0.80, *p* = .779). Therefore, due to the lack of Time X Protocol interactions, both protocols will be analyzed together for further predictor analyses. These outcome results indicate a significant reduction of OCD symptom severity as well as a reduction of depressive symptoms from baseline to post-treatment for both protocols (Fig. 1A+B). The course of the total treatment is represented in Fig. 1C+D.

### Response predictors

#### Baseline characteristics

At baseline no differences were found between responders and non-responders for age (F(1,59) = .97, *p* = .328), sex (χ^2^(1,n = 61) = .90, *p* = .343), medication use (χ^2^(1,n = 61) = 2.82, *p* = .093), OCD-symptom severity (F(1,59) = 2.98, *p* = .09) and MDD symptom severity (F,1,57) = 2.37, *p* = .129).

#### Sleep as predictor for treatment outcome

To select different sleep variables as potential predictors for treatment outcome, One-Way ANOVAs were performed to determine whether there are any differences in sleep parameters for responders vs. non-responders, summarized in Table 2.

**Table 2:** Differences between Responders and Non-Responders on sleep variables, with Cohen's effect size (*d*) as well as correlations between sleep variables and percentage improvement on YBOCS and explained variance (R^2^).

	Response		Correlation	
	*p*	*d*	R	R^2^
PSQI (global score)	.005	.825	-.305	.093
Subjective Sleep Quality #	.006	.862	-.337	.114
Sleep Latency #	< .001	1.086	-.413	.171
Sleep Duration	.401	.249	-.073	.005
Habitual Sleep Efficiency	.215	.378	-.211	.045
Sleep Disturbances	.845	.066	-.004	.000
Use of Sleep Medication	.312	.299	-.162	.026
Daytime Dysfunction #	.014	.748	-.379	.144
HSDQ (total)	.428	.224	-.099	.010
Insomnia #	.058	.522	-.209	.044
Parasomnia	.195	.355	.130	.017
CRSD*	.219	.332	-.201	.040
Hypersomnia	.994	.002	-.144	.021
RLS/PLMD*	.263	.300	-.003	.000
SBD*	.223	.331	-.122	.015
Actigraphy				
Bedtime (time)	.158	.439	-.221	.049
Get Up Time (time)	.484	.217	-.133	.018
Time In Bed (hours)	.419	.290	-.263	.069
Total Sleep Time (hours)	.193	.395	-.122	.015
Sleep Onset Latency (minutes)	.296	.314	-.167	.028
Sleep Efficiency (%)	.239	.369	-.62	.384
Wake After Sleep Onset (minutes)	.113	.496	.207	.043
Awakenings	.178	.410	-.004	.000

*CRSD = Circadian Rhythm Sleep Disorder, RLS/PLMD = Restless Leg Syndrome/ Periodic Limb Movement Disorder, SBD = Sleep Related Breathing Disorder; # indicates parameter included in response prediction (Cohen's *d*>0.5)

### Response prediction

Sleep parameters with an effect size *d*>0.5 were defined as potential predictors (see Table 2, marked #). PSQI (global score) correlated strongly (r>.7) with the four other variables, thus due to high collinearity, this variable was not used in the model. The remaining variables demonstrated no signs of collinearity with all r<.561. A discriminant model based on PSQI subscales 1) Subjective Sleep Quality (SSQ), 2) Sleep Latency (SL), and 3) Daytime Dysfunction (DD), and 4) HSDQ insomnia was carried out. This model could accurately predict rTMS response (Λ = .687, *p* = .005) and revealed an area under the curve (AUC) of .813 with a sensitivity of 76.0% and a specificity of 50.0%. The negative predictive value (NPV) was 45.5% and the positive predictive value (PPV) 79.2% yielding a normalized PPV of 1.51, suggesting an improved response rate of 51% when these sleep measures would have been a-priori used to select patients for rTMS treatment.

Finally, the CRSD-Model (Λ = .993, *p* = .922) and Insomnia-Model (Λ = .846, *p* = .146) as originally reported by Donse et al. (2017) were not significant and could thus not be replicated.

## Discussion

Here we report results from an open-label effectiveness study in 61 patients with therapy resistant OCD, treated with CBT and SMA rTMS or SMA-rTMS and prefrontal TMS aimed at the DLPFC to address (comorbid) MDD symptoms. Results demonstrated an overall clinical response rate of 53%, with no significant interactions between SMA rTMS only or including DLPFC augmentation, suggesting that response trajectories for primary OCD symptoms and comorbid MDD symptoms were rather comparable. These results complement results from recent controlled efficacy trials such as summarized in the Fitzsimmons et al. (2022) meta-analysis, where clinical efficacy was reported most specifically for the SMA and DLPFC protocols, with medium effect sizes. We found that rTMS and CBT can be effectively combined simultaneously in this treatment resistant population, with a response rate of 60% in patients with OCD and of 42% in patients with OCD and depression. Since a large effect size was found in this study compared to moderate effect sizes in studies using rTMS only (Fitzsimmons et al 2022), combining CBT and rTMS may be more effective than rTMS alone for OCD.

In line with Donse et al. (2017), non-responders showed a higher degree of sleep disturbances before treatment compared to responders. We found that a lower subjective sleep quality, a longer sleep latency, daytime dysfunctioning and insomnia as indexed by HSDQ resulted in less response to rTMS and CBT. The insomnia prediction model we developed suggests that response rate would have improved from 52.5% to 79.2% (51% improvement) if the model was used to select patients for treatment. Our earlier finding that CRSD predicted non-response to rTMS in patients with OCD (Donse et al., 2017) was however not replicated in this sample. A possible explanation might be the larger sample size analyzed in this study (n=61) relative to the small prior sample size (n=22).

Donse et al. (2017) found that OCD patients compared to non-OCD controls reported more frequently symptoms of insomnia, parasomnia, Circadian Rhythm Sleep Disorder (CRSD), hypersomnia, RLS/PLMD, and SBD. They also reported a lower sleep quality, longer sleep onset latency, lower habitual sleep efficiency and more daytime dysfunction compared to healthy controls, in line with other studies suggesting that sleep is frequently disturbed in patients with OCD (Nota et al., 2015; Paterson et al., 2013). Delayed bedtime for instance, a later bedtime than is typical or is desired, has been associated with more severe OCD symptoms compared to individuals with earlier bedtimes (Schubert et al., 2019). Delayed bedtime also predicted prospective increase in both obsessions and compulsions in OCD patients but not healthy controls (Schubert et al., 2019). There are indications that lower subjective sleep quality leads to worsened symptom severity the next day in OCD (Naftalovich et al., 2021). Finally, it has been shown that sleep disturbances in OCD, such as delayed bedtime are associated with diminished response to exposure and response prevention (ERP) without rTMS (Coles et al., 2021), which could partly explain the association of treatment response with a lower subjective sleep quality and longer sleep latency in our study.

In rTMS treatment, the faciliatory role of sleep in neural plasticity has been hypothesized to increase long-term potentiation of rTMS (Zhang et al., 2017). However, the impact of pretreatment sleep disturbances on rTMS (without CBT) treatment outcome in psychiatric disorders is not well described in the literature and largely based on subjective questionnaires (Centorino et al., 2020). Both the absence and presence of sleep disruption have been associated with improved treatment response (Lowe et al., 2013; Rosenquist et al., 2013).

The effect of sleep disturbances on OCD symptomatology and treatment response might be explained by decreased executive functioning, such as inhibitory control which is frequently described in OCD (De Wit et al., 2012). Decreased inhibitory control could lead to more frequent intrusive thoughts and compulsive behaviors (Norman et al., 2016; Van Velzen et al., 2015). It was found that inhibitory control was negatively affected by sleep disruption in OCD (Nota et al., 2016). Treating this sleep disruption by realigning circadian rhythms with agomelatine, a melatonergic agonist/5HT_2c_ antagonist, was shown to reduce OCD severity (Coles & Goodman, 2020). Consequently, sleep disturbances could decrease inhibitory control and worsen obsessions and compulsions, rendering ERP less effective (Coles et al., 2021). Additionally, it has been shown that sleeping soon after ERP enhances consolidation and generalization (Pace-Schott et al., 2012), suggesting that impaired sleep after sessions could impair this learning process.

Although this study has several important strengths, such as the large sample size, as well as sleep-predictors for rTMS treatment response in OCD, it should be noted that this study also has several limitations. While this study mainly pertains an open-label effectiveness study, results should generalize well to a clinical setting. On the other hand, no strong conclusions can be drawn on protocol-specific effects due to the lack of randomization and control over placebo effects. Moreover, as subjects received both rTMS and CBT, no TMS-specific results can be extracted, and sleep predictors could in fact be more related to CBT response. However, the main goal of this study was to investigate predictors of response to rTMS in a clinical setting often combined with CBT, with a focus on sleep parameters, following-up on our earlier work by Donse et al. (2017). Decisions about MDD comorbidity and TMS targets chosen (SMA vs SMA+DLPFC rTMS) were made, according to clinical practice, based on TMS clinicians’ personal assessments. Information about exact dosing regimens and changes during treatment were also not tracked systematically. Nonetheless, differences in target distribution and their effect on treatment outcome using the double protocol are estimated to be minimal.

In our sample we included patients that underwent at least 10 sessions of rTMS, while some studies indicate that at least 20-30 (McClintock et al., 2018; Schulze et al., 2018) or even more sessions are needed to optimize the effect (Yip et al., 2017). This could imply that patients who dropped out after 10 sessions could have still responded to the rTMS if they had received more sessions. We decided to include this group (early drop-outs) in our analysis as not to overestimate the beneficial effects of rTMS, more accurately reflecting clinical practice.

Our findings suggest that the addition of CBT targeting insomnia (CBT-i) or sleep hygiene should be more frequently considered in clinical practice as part of OCD treatment, especially given its lack of side effects and cost effectiveness and given the transdiagnostic nature of sleep problems in psychiatry (Arns et al., 2021). Targeting sleeping problems before or during treatment for OCD may especially be of importance for treatment resistant patients to enhance treatment efficacy. In case of improvement, our next question would be if resolving sleep maintenance problems by using CBT-I could improve OCD symptoms by itself. Since non-responders experience more sleep problems at baseline compared to responders, it is also worth to question if resolving sleep problems in these patients make the patient more likely to respond to rTMS.
